# Checking Different Video Game Mechanics to Assess Cognitive Abilities in Groups with and without Emotional Problems

**DOI:** 10.3390/jintelligence12010001

**Published:** 2023-12-20

**Authors:** Francisco J. Román, Pablo Gutiérrez, Juan Ramos-Cejudo, Pedro Antonio González-Calero, Pedro Pablo Gómez-Martín, Cristina Larroy, Ramón Martín-Brufau, Carlos López-Cavada, María Ángeles Quiroga

**Affiliations:** 1 Department of Biological and Health Psychology, Autonomous University of Madrid, 28049 Madrid, Spain; 2 Department of Software Engineering and Artificial Intelligence, Complutense University of Madrid, 28040 Madrid, Spain; pabgut02@ucm.es (P.G.); pagoncal@ucm.es (P.A.G.-C.); pedrop@fdi.ucm.es (P.P.G.-M.); 3 Department of Psychology, Camilo José Cela University, 28692 Madrid, Spain; jramos@ucjc.edu; 4 Mind Group Ltd., 28008 Madrid, Spain; 5 Department of Personality, Assessment and Clinical Psychology, Complutense University of Madrid, 28223 Madrid, Spain; clarroyg@ucm.es; 6 Unidad de Corta Estancia, Hospital Psiquiátrico Román Alberca, National Service of Health, 30120 Murcia, Spain; ramonmail@gmail.com; 7 Behavior, Emotions, and Health Research Group, Department of Psychology, Universidad Europea de Madrid, 28670 Madrid, Spain; carlos.lopez@universidadeuropea.es; 8 Department of Social, Work and Differential Psychology, Complutense University of Madrid, 28223 Madrid, Spain; m.angelesquiroga@psi.ucm.es

**Keywords:** video games, abstract reasoning, visuospatial reasoning, perceptual speed, groups with emotional problems and without emotional problems

## Abstract

This study assesses cognitive abilities through video games for entertainment (Blek, Edge, and Unpossible) that were programmed from scratch to record players’ behavior and the levels achieved in a sample without emotional problems and in one with emotional problems. The non-emotional-problem sample was recruited from three universities and two bachelor’s degree programs. The emotional-problem sample was recruited from two outpatient centers. The participants in the emotional-problem sample completed reduced versions of the ability tests and video games, as required by their emotional problems. Three subtests of the Differential Aptitude Test that assessed abstract reasoning, visuospatial reasoning, and perceptual speed were selected as ability tests. All participants were required to complete a mental health questionnaire (PROMIS) and a brief questionnaire on their gaming habits and previous experience with the video games used. The results that were obtained showed good convergent validity of the video games as measures of cognitive abilities, and they showed that the behavior of players in the sample without emotional problems while playing predicted the level achieved in the Blek and Unpossible game fragments, but this was only true for Unpossible in the emotional-problem sample; finally, shorter versions of the Blek and Edge game fragments can be used because they maintain their good psychometric properties.

## 1. Introduction

The general factor of intelligence is usually estimated with batteries of intelligence tests. However, since its conception (Spearman 1904), it has been emphasized that the g-factor can be estimated using different measures. The principle of indicator indifference (Jensen 1998; Spearman 1904) describes how any task of cognitive performance (of whatever type, nature, and level) reflects the effect of intelligence (in a different way); therefore, intelligence can be measured in many ways. Quiroga et al. (2016) suggested that video games can be used to measure cognitive abilities. Several studies have found a medium-high correlation (0.40–0.70) between video game performance and performance on tests of cognitive abilities when using video games for entertainment (Baniqued et al. 2013; Ventura et al. 2013; Shute et al. 2015; Buford and O’Leary 2015; Foroughi et al. 2016; Kranz et al. 2017; Kokkinakis et al. 2017; Lim and Furnham 2018) and video games made specifically to measure cognitive processes or abilities (McPherson and Burns 2007, 2008). Therefore, video game performance can be considered an excellent approximation for the estimation of intellectual level, and it has certain advantages over tests: (a) It takes place in a more natural environment (Koch et al. 2021), (b) it is highly accepted by the tested persons (Buil et al. 2020), and (c) is especially useful when anxiety can affect the validity of the tests (Ventura et al. 2013), as could be the case with young adults with emotional problems who consult outpatient centers (Bear et al. 2020).

The medium/high association between cognitive measures and video game performance can be explained by the following: (a) Some video games raise new problems to solve for the players and (b) using several cognitive processes is necessary to solve video games. For example, even though all video games are visuospatial, some require more visuospatial ability (such as Splatoon^®^), others require more planning (such as in Portal 2^®^), others require more perceptual speed (such as in Sky Jump^®^), etc. Puzzle-type video games (e.g., Portal 2^®^, Big Brain Academy^®^) were employed in most of the mentioned studies. However, some MOBAs (Multiplayer Online Battle Arena games) have also been analyzed (see Kokkinakis et al. 2017). Recently, Simons et al. (2023) examined the feasibility of a virtual reality video game about job performance to assess intelligence during personnel selection. Therefore, previous results showed that the two types of instruments (video games and tests) similarly sorted out individual differences among people. That is, cognitive performance can be predicted from video game performance. However, not all video games serve as an appropriate tool for intelligence or cognitive performance. Quiroga et al. (2009) described the characteristics that video games must have so that intense and/or extensive practice with them does not make their execution automatic, i.e., that they are learned and, thus, no longer serve for evaluating differences in cognitive performance. In general, the video games used in these studies mentioned had three characteristics: (a) moderate levels of complexity, (b) low consistency across training blocks, and (c) no possibility of transferring previous skills.

In 2015, Quiroga et al. explored the possibility of designing a battery of video games to assess several different abilities from the second stratum as a battery of tests similar to the Wechsler scales. Using confirmatory factor analysis techniques, Quiroga et al. (2015) showed that a battery of cognitive video games (“brain games”) selected from the Big Brain Academy^®^ video game reproduced five of the eight second-stratum factors of the hierarchical Cattell–Horn–Carroll model of intelligence (McGrew 2009; Schneider and McGrew 2018). In addition, the latent factor of general ability estimated with the video game battery showed a high correlation (*r* = 0.93) with the latent factor of general intelligence estimated with a battery of tests. This result implied that evaluations with video games (“brain games”) sort individuals in the same way as that of intelligence and ability tests, opening the possibility for developing video game batteries that assess interindividual differences in intelligence and ability. Nevertheless, brain games are very similar to laboratory tasks and tests designed to assess cognitive abilities and processes, so the high correlation obtained in the study by Quiroga et al. (2015) may be partially due to this. To test this hypothesis, Quiroga et al. (2019) developed a battery of video games of different genres (shooters, platforms, puzzles, and sports) whose performance was correlated with that on a battery of tests. The results showed medium and high correlations between both instruments (video games and tests), supporting the convergent validity of video games from different genres as measures of cognitive abilities. Moreover, the general latent factor of cognitive performance obtained with video games again showed a high correlation (*r* = 0.79) with the latent factor of general intelligence. Therefore, “non-brain games” video games can also be used to assess individual differences in cognitive abilities in a way similar to that of tests.

Video games designed to entertain are, however, of reduced usefulness for researchers and applied educators in their standard format because they do not provide speed and achievement scores that allow an accurate description of a player’s performance or the process data of how the game is being played (Quiroga et al. 2016). In general, video games for entertainment provide only the level attained in the game (score or level) and, sometimes, the time spent. However, suppose that some video games for entertainment are reprogrammed to maintain their cognitive requirements. In that case, this opens the possibility of recording the variables of execution in the game (e.g., the time to first response, type of action carried out, degrees in which it was turned, and so on), making it possible to calculate performance in the game and to know how the behavior of the player while playing contributes to that performance. This task requires the collaboration of computer scientists (who reprogram the games) and educators and psychologists (who conceptually analyze the games and their requirements to design which aspects of the player’s performance should be recorded and at which moments of the game).

Using this approach, Guo et al. (2020) selected three video games (Blek, Edge, and Unpossible) from those used by Quiroga et al. (2019) because they presented good correlations with the cognitive abilities that they estimated (abstract reasoning, visuospatial reasoning, and perceptual speed) and were novel for the players. Guo et al. applied machine learning to forecast a player’s final score in a game using data obtained from a telemetry system. This telemetry system meticulously recorded every in-game action performed by the player while machine learning algorithms analyzed these data to predict the player’s overall performance. Remarkably, the study explored the possibility of making accurate predictions about a player’s final score by examining data from the initial minutes of each gaming session. Even if players were not given enough time to reach their peak performance, the study demonstrated that it was still feasible to predict their ultimate in-game performance with a high degree of accuracy without introducing significant errors. This practical insight is particularly valuable because it can streamline the process of intelligence assessment, making it quicker and more user-friendly, aligning with one of the central goals of this research. Different machine learning algorithms were used to predict the final performance of players in each of the games based on truncated traces, meaning that these temporal sequences were considered up until a certain point in time. When using half of the original experiment time as a cut timestamp, the methods employed were able to predict the final level in Blek with an average error of approximately three levels (out of 26 levels in the game), that in Edge with an average error of around 0.9 levels (out of 8 levels in the game), and the final number of deaths in Unpossible with an average error of about 1.58 deaths (in practice, this value oscillated between 3 and 25 deaths per player). This demonstrated that the difference between the predicted and actual outcomes remained within the range of 5–10% in error, notwithstanding a substantial 50% reduction in the experiment’s duration, which suggested that the reduction in the experiment’s duration did not lead to a significant loss of information.

Once a game is reprogrammed, it is essential to understand what roles the different response variables play in the level achieved. This is not a matter of adding the response variables to the achieved level but of understanding why some people do not solve certain video games. The aim of this study was to understand which response process variables allowed us to understand the variability in the achieved level variable. Understanding these variables would help to understand the following:

1. From the side of video games: how to modify game mechanics (e.g., implementing difficulty levels).

2. From the side of individuals: Why do people with similar abilities not play video games in the same way?

This study aims to (1) analyze the psychometric properties (internal consistency and convergent validity with respect to ability tests) of each of the three reprogrammed versions of the video game mechanics from Guo et al. (2020), (2) analyze whether the performance variables in each video game predict the level achieved, and (3) analyze the minimum playing time necessary to obtain a reliable and valid score about the cognitive ability assessed with each video game. These three aspects were assessed in groups of people who differed in their levels of mental health: typically developing young adults and young adults with emotional problems who consulted outpatient centers. Analyzing these three aspects together with emotional problems in community samples is a novelty concerning the work in this area. The population with emotional problems is one of those that can benefit the most from having video games available to assess their cognitive characteristics, since they are usually assessed repeatedly and, in some cases, show reluctance (Boot 2015; Koch et al. 2021). In addition, emotional problems often decrease test performance (Hopko et al. 2005; Keyes et al. 2017), and video games may be an alternative that measures more accurately in this population, since video games have greater acceptance by the persons evaluated (Buil et al. 2020) and video game performance sorts individuals in the same way as that of intelligence and ability tests (Quiroga et al. 2015). Moreover, video games can include different emotional content, allowing the same cognitive aspect to be assessed in the face of different emotions (Villani et al. 2018).

## 2. Materials and Methods

### 2.1. Participants

The non-emotional-problem sample consisted of 97 participants recruited from three universities in Madrid (Universidad Complutense de Madrid—UCM, Universidad Autónoma de Madrid—UAM, and Universidad Europea de Madrid—UEM).

The emotional-problem sample consisted of 21 participants from two outpatient psychology clinics in Madrid: the University Clinic of the UCM (CUP-UCM) and the Mind Group Clinic (MIND). The CUP-UCM is a health center for psychological care that also has teaching and research among its purposes. It treats people from inside and outside the university community, although most patients (approximately 45%) are university students at UCM. The mental health problems most frequently treated at the CUP-UCM are anxiety and mood problems. However, a wide range of problems are treated (self-esteem, family problems, adjustment problems, personality problems, bullying, violence).

Mind Group Ltd. is a private clinic for psychotherapy in downtown Madrid. The Mind Group Clinic mainly treats patients from Madrid (Spain). It offers patients tailored CBT and DBT treatments and applies multiple evidence-based therapy methods, which are mainly for mood disorders, anxiety disorders, and personality disorders. 

#### 2.1.1. Recruitment

In the three universities that participated in the study, posters that included a generic description of the study were posted so as not to bias participation concerning the main goal of the study; they presented statements such as: “We are conducting a study to analyze the (cognitive) abilities required by various video games. We would like to count on your participation. If you are interested, please contact …”. The recruitment messages specified that participation in the study would not influence the students’ grades. Once a participant showed willingness to participate and signed the informed consent form, they were called to a group evaluation session. 

In the clinics, an evaluation was offered for free, and an anonymized individual report was given to each participant as thanks for their participation.

#### 2.1.2. Exclusions

As an inclusion criterion, participants in the non-emotional-problem sample were required to have enough time (two hours) to complete the video games and tests. Three participants who did not complete the two hours of evaluation were excluded from the analysis (incomplete data). A general mental health questionnaire (PROMIS total—Patient Reported Outcome Measurement Information System, Vilagut et al. 2015) was used to verify the mental health status of the participants to be included in both the non-emotional-problem and emotional-problem groups. In addition, for the emotional-problem sample, psychotic disorders and neurological conditions were excluded.

#### 2.1.3. Final Sample

From UCM, 45 students from the School of Computer Science participated (46%); from UAM, 33 students from the School of Psychology participated (34%); from UEM, 19 students from the School of Biomedical Sciences participated (20%). The mean age of the participants was 22 years (standard deviation: 3.74). Of these, 46 were male (47.4%). Of the group evaluated, 82% were between 20 and 30 years of age, which is the age of the population that plays video games the most in Spain (Statista 2023).

For the emotional-problem sample, 13 patients from CUP-UCM and eight from MIND participated. The mean age of this group was 25.71 (SD = 5.41). Of these, 43% were male.

### 2.2. General Study Design

The participants completed three video games, three tests, and a mental health questionnaire. The order of completion was always the same: (1) informed consent about the study and transfer of data to the research team; (2) general health questionnaire; (3) cognitive ability tests; (4) video games.

### 2.3. Materials and Instruments

#### 2.3.1. Cognitive Ability Tests

For all groups, three subtests of the Differential Aptitude Test-5 Level 2 (Bennet et al. 2011) were used: the PSA (perceptual speed), AR (abstract reasoning), and SR (spatial reasoning) subtests. The version for online administration (DAT Next Generation, Pearson 2019) was used for the non-emotional-problem group. The participants in the emotional-problem group individually completed the AR and SR subtests from the paper-and-pencil screening version and the full DAT-PSA. These tests have proven their good psychometric properties (Pearson 2019).

#### 2.3.2. Video Games

The 3 video games programmed by Guo et al. (2020) were used to record the players’ behavior while playing them, in addition to the final level reached. For each of them, the level of previous experience of each player was also recorded. The non-emotional-problem group completed the full version. For the emotional-problem groups, a reduced version of Blek and Edge was developed, which, while maintaining high reliability (ω = 0.97 for both of them), required only half the time: 5 min for Blek and 6 min for Edge. The third video game (Unpossible) lasted 5 min for both groups. On both sites, after registering, the participant had to describe their knowledge of the game on a scale of 1 to 10. In addition, to characterize them as gamers, they answered these two questions: (1) the hours per week that they played video games and (2) the experience level they thought they had from 1 to 7. Moreover, the participants reported their level of experience with each game in the study using a 7-point Likert scale. The version of the video games used for this study may be delivered free of charge by requesting it from pabgut02@ucm.es.

Below is a summary of each video game and the recorded performance variables.

Blek: This was presented as a canvas on which the player drew (with the mouse on a PC) a short line or stroke that came to life, repeating itself in a loop until it went out of the screen or hit an obstacle. At each level, there was a set of circles or colored balls that the line needed to pick up as it moved across the screen. This game belongs to the puzzle category and is desirable for evaluating fluid reasoning (Gf). A game time of 10 min was set, as in previous works (Quiroga et al. 2019—commercial version of the video game—and Guo et al. 2020—reprogrammed version of the video game mechanics). The essentials of this video game are available at the following link: 

https://www.youtube.com/watch?v=D0YtH4ftRrw (accessed on 13 December 2023).

For each level of the game, we recorded the moment when the player touched the screen for the first time after a static period (first touch), the start of a drawing of a stroke (begin drawing), the start of the repetition phase of the stroke (begin looping), and collisions with obstacles in the game (black touched). From these response variables, the level that was overcome, the number of curves drawn until the level was overcome, the time spent thinking before drawing a new curve, and the total time spent solving the level were computed for each minute of play.

Edge: This consisted of several levels in which the player controlled a rolling cube that could move in 4 possible directions. The world had an isometric perspective composed of discrete squares along which the cube advanced. The player’s objective was to reach the final square of the level in the shortest time possible while collecting a set of prisms distributed all over the map. The solid spatial component of this game makes it an appealing candidate when it comes to evaluating a player’s visuospatial ability (Gv). A game time of 12 min was set, as in previous works (Quiroga et al. 2019—commercial version of the video game—and Guo et al. 2020—reprogrammed version of the video game mechanics). The essentials of this video game are available at the following link:

https://www.youtube.com/watch?v=xGY0LKSJHpw and ab_channel=Mobigame (accessed on 13 December 2023).

For each level of play, the following variables were recorded: collectible prisms obtained (got item), new progress marks reached (got checkpoint), and an additional parameter (num moves) at the end-of-level events to provide the total number of movements made in the level. From these response variables, the level that was overcome, the number of prisms collected, and the number of checkpoints that were overcome were computed for each minute of play. The number of moves could not be calculated for each minute of play, which was a reason for why they were excluded from the analyses in this study.

Unpossible: Unlike the other two games, the mechanics of Unpossible were more straightforward: The player rode on the outside of a curved tube in space and tried to hold on as long as possible by dodging all of the obstacles that they encountered. The player did not control the forward movement; they could only turn left or right on the tube. Every time players hit an obstacle, they died and were respawned at the beginning of the corresponding level. As the game progressed, the sequence of obstacles became more complex and demanding, requiring the player to react faster and constantly concentrate on controlling their movements. Therefore, this is a very up-and-coming candidate for measuring the processing speed (Gs) and probably a certain amount of visuospatial ability (Gv). A game time of 5 min was set, as in previous works (Quiroga et al. 2019—commercial version of the video game—and Guo et al. 2020—reprogrammed version of the video game mechanics). The essentials of this video game are available at the following link:

https://www.youtube.com/watch?v=XBafeyTwq6Y (accessed on 13 December 2023).

In this game, no additional events were considered (beyond the start and the end of the test and the number of attempts). However, new parameters were added to the player death events with information about the turns and keystrokes made by the user in each direction. Similarly, new parameters were added to determine the point on the curve where the player died in the corresponding attempt. 

Two variables were calculated for each attempt to stay in the tube: keystrokes per second and degrees of rotation per keystroke, that is, how many degrees the player angularly traversed and how many times they pressed a spin key during a given attempt. These variables provided information about the player’s playing speed and the efficiency of the moves made.

Log Data: A telemetry system was designed to collect players’ interactions with the games for data recording. This system was based on events, making it versatile and adaptable to games and instrumented applications. Each event included basic mandatory information, such as the game and user identifiers that generated it, a timestamp in UNIX format, a representative name of the event, and a dictionary or list structure for additional parameters that helped contextualize and manage the events (as an example, in a level start event, such a parameter could be the identifier of the level in question).

Priority was given to ease of use and clarity in implementing the telemetry system in the game code. The aim was that programmers could send events following an intuitive logic similar to the one used in telemetry systems that are in widespread use, such as Unity Analytics. Moreover, the developed code is reusable, which means that it can be applied to any other game implemented in the same engine without additional adaptations.

The main goal of the telemetry system was to collect detailed data on players’ interactions with the games, allowing further analysis to obtain different metrics for calculating execution and performance variables in the video game.

#### 2.3.3. General Mental Health Questionnaire

The Patient-Reported Outcome Measurement Information System (PROMIS; Vilagut et al. 2015) was used in its Spanish version with the Sleep, Anxiety, and Depression scales.

The Patient-Reported Outcome Measurement Information System—Depression domain (PROMIS-D; Cella et al. 2007) is an 8-item self-reported measure that appraises the severity of depressive symptomatology (e.g., “I felt that I have nothing to look forward to”). Responses range from 1 = “Never” to 5 = “Always”, so the total score varies from 8 to 40. Previous findings showed good psychometric properties and validity in the original and Spanish versions of PROMIS (Cella et al. 2007; Vilagut et al. 2015). 

The Patient-Reported Outcome Measurement Information System—Anxiety domain (PROMIS-A; Cella et al. 2007) is an 8-item self-reported measure that appraises the severity of anxious symptomatology (e.g., “I felt nervous”). Responses range from 1 = “Never” to 5 = “Always”, so the total score varies from 8 to 40. Previous findings showed good psychometric properties and validity in the original and Spanish versions of PROMIS (Cella et al. 2007; Vilagut et al. 2015). 

The Patient-Reported Outcome Measurement Information System—Sleep domain (PROMIS-S; Cella et al. 2007) scale comprises four items that were developed to assess sleep disturbances in the last seven days. The items include the following: “My sleep quality was…”; “My sleep was refreshing…”; “I had a problem with my sleep…”. Each item has a 5-point response scale. The response options for the sleep quality item range from “very poor (1)” to “very good (5)”, and for the remaining items, they range from “not at all (1)” to “very much (5)”. This short form was initially developed using a population with a greater prevalence of chronic illnesses. 

### 2.4. Data Analysis

The following analyses were carried out for each objective: 

Objective 1: Pearson’s correlations were computed to analyze the relationship between the game’s achievement variables and cognitive abilities.

Objective 2: We conducted a stepwise regression analysis for each video game separately to analyze whether the game execution variables predicted the level achieved. 

Objective 3: A stepwise regression analysis was computed separately for each minute of play and video game to analyze the minimum playing time that was necessary to obtain a reliable result about the cognitive ability assessed by each video game. 

For all analyses, the SPSS 27 program was used. The confidence level was set to α = 0.05. The power achieved for the different analyses was estimated using G*Power software version 3 (Heinrich-Heine-Universität Düsseldorf, Düsseldorf, Germany).

## 3. Results

Table 1 shows the descriptive data of the two samples and the PROMIS health questionnaire, broken down according to scales and comparative statistics. The two groups evaluated (those without and with emotional problems) did not statistically differ in terms of sex distribution, age, and self-perceived expertise. However, the non-emotional-problem group spent a greater number of hours per week playing video games than the emotional-problem group did (9.10 vs. 6.89, *t* (114) = −3.67, *p* < .001, *d* = 14.57). Regarding the PROMIS health scales, there were no statistically significant differences in any of the three scales. However, on the depression scale, they bordered on statistical significance (*p* = .07), with the emotional-problem group showing a score that was more than two points higher. This aspect corroborates that they requested help in the respective clinics for emotional problems.

Table 2 shows the descriptive data for the two groups regarding the video games and the tests of cognitive abilities. It should be remembered that the averages could be compared because the emotional-problem group completed reduced versions of all of the tasks except for the DAT-PSA. In this test, the emotional-problem group’s data showed a lower perceptual speed that bordered on statistical significance (*t* (116) = 3.68, *p* = .054). In both groups, most participants were unaware of the video games that they would play: (1) non-emotional-problem group: Blek—92%, Edge—90%, and Unpossible—97%; (2) emotional-problem group: All participants were unaware of the games. 

Concerning the hours/week spent playing and experience as video gamers, the two groups were similar in both aspects (hours per week: *t* (114) = 0.60, *p* > .05; video game expertise: *t* (114) = −1.43, *p* > .05). However, as shown when employing the hours/week spent playing by each group, the non-emotional-problem group spent, on average, 3 h more per week, but both groups were very heterogeneous, although they were equally heterogeneous (hours per week: F *Levene* = 0.67, *p* > .05; video game expertise: F *Levene* = 0.02, *p* > .05).

The Blek and Edge video game scores showed high reliability (calculated using the ω index) for all of their scores in both the non-emotional-problem sample [Blek (level = 0.99, total curves = 0.99, and mean time thinking = 0.98); Edge (level = 0.98, prisms = 0.99, and checkpoints = 0.99)], and in the emotional-problems group [Blek (level = 0.93, total curves = 0.99, and mean time thinking = 0.90); Edge (level = 0.82, prisms = 0.95, and checkpoints = 0.92)].

The results obtained for each objective are shown separately below.

### 3.1. Objective 1: The Relationship between the Game’s Achievement Variables and the Assessed Cognitive Abilities

Table 3 shows the correlation values obtained between the ability test scores and the achievement in each video game for the two samples. The 95% confidence interval is also included.

In both samples (without and with emotional problems), the three video games were correlated with abstract reasoning (DAT-AR) and visuospatial reasoning (DAT-SR). However, in the non-emotional-problem sample, the video game Unpossible was not correlated with perceptual speed (DAT-PSA). The video game Unpossible was correlated with abstract reasoning and visuospatial reasoning in the emotional-problem sample. These results support, in both samples, the convergent validity of the Blek and Edge video games. The Unpossible video game showed convergent validity as a measure of perceptual speed only in the emotional-problem sample.

### 3.2. Objective 2: Do the Variables of Game Execution Predict the Level Achieved?

The obtained results are included in Table 4 for the non-emotional-problem sample. For the Blek and Unpossible video games, the stepwise regression analysis showed two predictive models. For the Blek game, drawing fewer curves and spending less time thinking predicted 26% of the variance in the final level overcome. For the video game Unpossible, combining a few keystrokes per second and a smaller number of degrees of a turn per keystroke predicted 30% of the variance in the final level overcome. For the video game Edge, the stepwise regression analysis showed only one model in which the variable checkpoints that were overcome predicted 96% of the variance in the final level reached. This high percentage showed that here was a high isomorphism (collinearity) between the variables of the checkpoints overcome and the final level overcome. The variable prisms collected did not predict any significant percentage of the final variance.

For the emotional-problem sample, the results are included in Table 5. In this case, the aggregate response variables did not predict the level reached for the Blek video game. For the Edge video game, the analysis provided only one model in which, as in the non-emotional-problem sample, the aggregate variable checkpoints overcome predicted 87% of the variance in the final level. For the Unpossible video game, the stepwise regression analysis showed three models. The most complete one included a standardized beta coefficient greater than one, so this model’s result needed to be revised. The second model showed fewer keystrokes per second and fewer degrees of rotation per keystroke, predicting 59% of the variance in the number of attempts required to stay five minutes on the tube in the video game. Therefore, a greater number of keystrokes per second and higher degrees of rotation per keystroke predicted a better result.

### 3.3. Objective 3: To Analyze the Minimum Playing Time Necessary to Obtain a Reliable and Valid Result Regarding the Cognitive Ability Assessed with Each Video Game

This objective was only studied for the non-emotional-problem sample in our study. The participants in the emotional-problem group played for a reduced amount of time due to the characteristics of their emotional problems (Blek: 5 min; Edge: 6 min). Concerning the video games, in Unpossible, the log data were not collected by time but by each attempt made by the player to stay on the tube without colliding with an obstacle, which made it impossible to calculate whether a game time of fewer than 5 min would be equally reliable and valid. The analysis, therefore, focused on the first two video games.

Table 6A shows the results obtained for the Blek video game. Considering the percentages of variance of the final level variable, which was predicted from the results for each minute, it was clear that shorter versions of the game time could be used. The minimum time could be four minutes (prediction of 70% of the variance in the final level overcome). However, this decision must be made by each researcher or person conducting the assessments depending on the objectives to be achieved: A shorter evaluation time (4 min, for example) will facilitate the evaluation of complex populations, while a longer assessment time (6 min, for example) will increase the confidence in the score obtained because it corresponds to a greater extent with the score that was obtained with the 10 min considered in this study. In the nine minutes into which the execution was decomposed, the two variables that predicted the final performance in the game were the level overcome in that minute and the thinking time (until starting the drawing of the curves). Appendix A includes the reliability (ω) and convergent validity (correlations with DAT-AR, SR, and PSA) of the scores obtained in each minute of play so that each researcher/person conducting such assessments can decide the most appropriate playing time for their case.

Table 6B shows the results obtained for the Edge video game. Considering the variance percentages of the final level overcome variable, which was predicted from the results for each minute, shorter versions of the game could also be used in this video game. In this case, the minimum playing time could be five minutes, since the level overcome in the game in that time predicted 70% of the final levels overcome. In the eleven minutes into which the execution was decomposed, the two variables that predicted the final performance in the game were the number of prisms collected and the number of checkpoints overcome. However, our results showed that both variables could be considered isomorphic, as we pointed out in the results for the previous objective. In fact, the checkpoints overcome were established and recorded (although they were not in the commercial version of the game) to consider the course completed at a level that was not overcome. Appendix B includes the reliability (ω) and convergent validity (correlations with DAT-AR, SR, and PSA) of the scores for the checkpoints overcome for each minute of play so that each researcher/person conducting such assessments can decide on the most appropriate playing time for each case. Appendix C and Appendix D include the outcomes in each game for the Bleck and the Edge video games.

## 4. Discussion

This study was designed to explore whether reprogrammed games for entertainment (Blek, Edge, and Unpossible) were proper tools for measuring intelligence in two samples: one with emotional problems and one without. The results obtained in this study are remarkable from several perspectives. We also tested the feasibility of video games for measuring intelligence in young patients undergoing outpatient psychological treatment because these patients are going through a stage with difficulties in managing their emotional state, which could affect their performance in standard measures of intelligence (Ventura et al. 2013).

First, the results confirmed the convergent validity of the mechanics of the reprogrammed versions of these games for entertainment as measures of cognitive abilities, as several researchers have shown for video games in general (Baniqued et al. 2013; Buford and O’Leary 2015; Foroughi et al. 2016; Kokkinakis et al. 2017; Kranz et al. 2017; Lim and Furnham 2018; McPherson and Burns 2007, 2008; Quiroga et al. 2019; Shute et al. 2015; Ventura et al. 2013). The correlation values obtained between test performance (DAT-AR and DAT-SR) and video game performance (Blek, Edge, and Unpossible) were of average size for the non-emotional-problem sample (0.37 to 0.46), and they were similar to values obtained in other studies when correlating tests that measured the same ability. For example, Gills et al. (2019) showed correlations of 0.55 between two versions of a declarative memory test and 0.44 between a declarative memory test and a perceptual speed pattern recognition test. Therefore, the correlation values obtained between performance in the three video games and scores in abstract reasoning (DAT-AR) and visuospatial reasoning (DAT-SR) reflected that the three video games ranked individuals in terms of their abstract and visuospatial reasoning ability in a way similar to that of the tests used. It is interesting to note that the three video games were correlated with both abilities (abstract and visuospatial reasoning), showing how these video games required both abilities to be solved. Regarding perceptual speed (measured with the DAT-PSA), only the Blek video game showed a statistically significant correlation. This result was similar to that obtained by Quiroga et al. (2019) and most likely indicates that the perceptual speed required in Unpossible is not identical to that assessed with the DAT-PSA in typically developing youngsters.

In the emotional-problem sample, the correlation values obtained between performance on the tests (DAT-AR and DAT-SR) and performance on the video games (Blek, Edge, and Unpossible) were also of medium size (0.36 to 0.53). Therefore, in the emotional-problem group, the correlation values obtained between performance in the three video games and scores in abstract reasoning (DAT-AR) and visuospatial reasoning (DAT-SR) reflected that the three video games ranked individuals in terms of abstract and visuospatial reasoning ability in a way similar to that of the tests used. In this emotional-problem group, the DAT-PSA was correlated with video game performance, indicating that in the group, the DAT-PSA captured the inter-individual differences in perceptual speed required by the video games. Thus, with outpatients with emotional problems, as a reason for consultation, video games can also be used to assess cognitive abilities. These results, which are pioneering in this area, are important because they provide an alternative assessment for populations that are reluctant to be assessed with tests, as has been proposed before (Boot 2015; Koch et al. 2021).

Secondly, the aggregate variables that reflected the response process for the Blek video game (total curves drawn and average time) only predicted a small percentage of the total variance of the final score of the video game in the non-emotional-problem sample and did not predict the final level reached in the game in the emotional-problem sample. That is, the variables that reflected the players’ response processes were more practical for understanding the execution at each moment of the video game (see Table 6) but not so much as aggregate variables for explaining the final level overcome. As these minute-by-minute response process variables have now been recorded, future work can establish player profiles using clustering procedures that show how players deal with game demands. In the case of the Edge video game, it would also be necessary to compute the number of moves or steps taken, since the variable of the number of checkpoints overcome is collinear with the variable of the levels overcome. Detecting the variables of the response process that are useful in each video game requires a further in-depth analysis of these games, as was also found in the study of Peters et al. (2021).

With the video game Unpossible, the variables of the response process (keystrokes per second and degrees of rotation per keystroke) could not be computed for the total time played, since, due to the characteristics of the video game, they were calculated per attempt to avoid falling off of the tube. Each player carried out a different number of attempts. Thus, some participants completed 5 min of play in two attempts, while others needed more than seven attempts. Because of this, only the response process variables were calculated for the first two attempts. The results showed that in the non-emotional-problems group, these variables predicted about 30% of the variance of the final level overcome. In the emotional-problem groups, these variables explained 64% of the variance of the final level overcome. This result shows that in this video game, a low perceptual speed is associated with a lower number of keystrokes per second (delayed reaction times) and a lower number of degrees of rotation per keystroke (erroneous estimation of the position to occupy). This result has an important practical application, as perceptual speed training is quite common in older people to slow cognitive decline (e.g., Edwards et al. 2010; Ball and Vance 2008; Hoffmeister et al. 2023). Knowing a participant’s level of dealing with game demands would help personalize their training in perceptual speed, and this would be possible with a five-minute assessment with the fragment of the reprogrammed Unpossible video game.

Thirdly, we studied the possibility of using shorter versions of the Blek and Edge video games, which maintained the reliability and validity of their scores. The results indicated that with 4 min of playing Blek and five minutes of playing Edge, a high prediction of the final variance is achieved while maintaining the reliability and convergent validity of the scores (see Appendix A and Appendix B). These results obtained in the non-emotional-problem group indicated that the playing times that we established for the emotional-problem group at the clinic’s request were adequate—5 min for Blek and 6 min for Edge. Shorter versions of tests and, in this case, of video games are essential for populations with emotional problems and populations that are reluctant to be tested or to be tested repeatedly—as shown, for example, by Afshar et al. (2021) for dementia, Estrada-Orozco et al. (2018) for mild cognitive disorder, and Walterfang et al. (2006) in neuro-psychiatric patients.

Taken together, the data from this study show that it is useful to use reprogrammed versions of commercial video game mechanics that automatically record player performance to the second, making it possible to compute performance scores by time segments and scores that reflect behavior while playing. Using games for entertainment has the advantage of their being attractive in their design and game mechanics. By reprogramming them, as in this study, they also function as tests that automatically provide the researcher/person conducting the assessment with the level achieved by the player, as well as the behavioral characteristics displayed while playing. This approach (reprogramming the mechanics of games for entertainment and recording performance variables) is, therefore, different from those that proposed using video games of the sandbox genre to program tests that are already known (Peters et al. 2021; Unzueta-Arce and Hidalgo-Muñoz 2022). It also differs from those who have opted to computerize tests by adding gamification elements (Mallancini et al. 2021).

Like all studies, this one also has limitations, which are essentially due to the small number of participants evaluated in the emotional-problem sample, which calls the stability of the results obtained into question, so it would be advisable to repeat this study with a number of participants similar to that in the non-emotional-problem sample. Another variable that may have affected the results was the level of experience with video games. In our study, both samples self-perceived themselves as having the same experience with video games. However, the results showed that, on average, the non-emotional-problem sample spent more time playing video games than the emotional-problem sample did. Furthermore, the emotional-problem sample was very heterogeneous, and some patients could have had a more severe diagnosis than what we identified. In addition, the PROMIS test results showed no significant differences between the emotional-problem group and the non-emotional-problem group. Perhaps it would have been better to use other questionnaires from the literature to better characterize the psychological problems of the clinical sample in therapy during the study. Regarding possible improvements derived from what was observed in this study, it would be necessary to record other behavioral variables while playing Edge, given that the variables recorded in this study showed high collinearity with the final level achieved. Finally, since a perceptual speed games—Unpossible—was reprogrammed, it would be interesting to include a forced rest when several failures occur in a row, thus avoiding non-reflexive behaviors while playing.

## 5. Conclusions

In short, this study shows that entirely reprogramming the mechanics of games for entertainment to record the players’ behavior together with the final level overcome allows scores that present high reliability and convergent validity to be used as measures of cognitive abilities. As such games are designed to entertain, they are attractive. However, they do not evaluate only one ability but require the analysis of three (abstract reasoning, visuospatial ability, and perceptual speed). It was also shown that the response process variables were relevant to understanding the performance of the emotional-problem group in perceptual speed. Finally, it was verified that shorter versions of the Blek and Edge video games could be used, as their scores maintained high reliability and acceptable convergent validity.

## Figures and Tables

**Table 1:** Descriptive and comparative data from the demographic data and the PROMIS health scales of Sleep (S), Anxiety (A), and Depression (D) for the two groups. VG = video games.

	Non-Emotional Problems Group			Emotional Problems Group			*t/χ* ^2^	*p*
	*N*	*M*	*SD*	*N*	*M*	*SD*		
Sex (females)	51	---	---	11	---	---	5.27	.153
Age	97	22.11	3.74	21	25.71	5.40	−1.43	.078
Video game expertise	97	3.15	1.91	21	3.84	1.97	0.60	.274
Hours per week playing VG	97	9.10	15.43	21	6.89	8.73	−3.67	<.001
PROMIS S	97	10.30	3.44	21	10.62	4.53	−0.36	.358
PROMIS A	97	20.19	67.69	21	21.48	5.74	−0.81	.209
PROMIS D	97	16.44	6.73	21	18.86	6.23	−1.51	.067

**Table 2:** Descriptive data for the two groups on the tests, video games and experience playing video games (VG).

**Non-Emotional-Problems Group**	***M***	***SD***	**Z Asymmetry**	**Z Kurtosis**
DAT-AR *	57.32	9.80	−1.84	−1.49
DAT-SR *	55.23	8.73	−1.10	−1.26
DAT-PSA **	69.07	14.69	−5.30	10.96
Video game expertise	3.16	1.91	−4.60	−2.58
Hours per week playing VG	9.10	15.43	16.18	40.35
Blek—final level overcome	14.18	6.26	1.02	−2.01
Blek—number of curves drawn	99.78	40.61	5.82	6.62
Blek—mean time thinking	456.18	48.84	−1.60	0.05
**Emotional-Problems Group**	***M***	***SD***	**Z Asymmetry**	**Z Kurtosis**
DAT-AR	10.48	3.82	−0.44	−0.55
DAT-SR	11.24	3.85	0.52	−0.25
DAT-PSA **	61.90	17.91	−1.21	−0.58
Video game expertise ***	3.84	1.98	0.73	−0.80
Hours per week playing VG ***	6.89	8.73	3.04	1.68
Blek—final level overcome	5.95	3.07	2.56	2.95
Blek—number of curves drawn	51.57	16.26	1.92	0.65
Blek—mean time thinking	226.19	14.59	−0.76	0.75

Note: * DAT—Next Generation was administered online to the non-emotional-problems group, and the scores are expressed as T-scores with a mean of 50 and SD of 10. ** Both groups completed the same paper-and-pencil version of this test. *** Two people did not respond to this question; *n* = 19.

**Table 3:** Pearson correlations between tests and video games for the two groups studied (95% CI).

	Non-Emotional-Problems Group (n = 97)			Emotional-Problems Group (n = 21)		
Video Game and Performance Variables	DAT-AR	DAT-SR	DAT-PSA	DAT-AR	DAT-SR	DAT-PSA
Blek	0.45 ***	0.39 ***	0.27 **	0.51 *	0.42	0.14
Levels overcome	[0.27 to 0.59]	[0.20 to 0.55]	[0.07 to 0.45]	[−0.28 to 0.56]	[−0.01 to 0.72]	[−0.31 to 0.54]
Edge	0.42 ***	0.37 ***	0.00	0.36	0.53 *	0.36
Levels overcome	[0.24 to 0.57]	[0.18 to 0.53]	[−0.20 to 0.20]	[−0.08 to 0.69]	[0.12 to 0.78]	[−0.08 to 0.68]
Unpossible	−0.47 ***	−0.42 ***	−0.08	−0.48 *	−0.45 *	−0.41
Number of attempts	[−0.61 to −0.30]	[−0.57 to −0.24]	[−0.28 to 0.12]	[−0.76 to −0.05]	[−0.75 to −0.01]	[−0.72 to 0.03]

Note: * *p* < .05; ** *p* < .01; *** *p* < .001.

**Table 4:** Final predictive models (stepwise regression analysis) obtained for each video game in the non-emotional-problems group.

Variable	β	R^2^	Adjusted-R^2^
**Blek**		0.26	0.24
Total curves drawn	−0.30 **		
Total time thinking	−0.30 **		
**Edge**		0.96	0.96
Checkpoints overcome	0.98 ***		
**Unpossible**		0.30	0.29
Keystrokes per second—first attempt	−0.55 ***		
Degrees of rotation per keystroke—second attempt	−0.24 **		

Note: ** *p* < .01; *** *p* < .001; statistical power range: 0.99–1.00.

**Table 5:** Final predictive models (stepwise regression analysis) obtained for each video game in the emotional-problem group.

Variable	β	R^2^	Adjusted-R^2^
**Blek**		---	---
Total curves drawn	---		
Total time thinking	---		
**Edge**		0.90	0.90
Checkpoints overcome	0.95 ***		
**Unpossible**		0.59	0.54
Degrees of rotation per keystroke—first attempt	−0.77 ***		
Keystrokes per second—first attempt	−0.44 *		

Note: * *p* < .05, *** *p* < .001; statistical power range: 0.98–1.00.

**Table 6:** Predictive models for (A) the Bleck and (B) Edge video games per minute of play.

	(A) Bleck		(B) Edge	
Variable	R^2^	Adjusted-R^2^	R^2^	Adjusted-R^2^
First minute	0.29	0.28	0.44	0.43
Second minute	0.51	0.50	0.60	0.50
Third minute	0.66	0.65	0.64	0.64
Fourth minute	0.71	0.70	0.68	0.67
Fifth minute	0.72	0.72	0.72	0.71
Sixth minute	0.84	0.83	0.77	0.76
Seventh minute	0.89	0.89	0.74	0.74
Eighth minute	0.94	0.94	0.70	0.78
Ninth minute	0.97	0.97	0.82	0.82
Tenth minute	---	---	0.87	0.87
Eleventh minute	---	---	0.91	0.91

Note: Statistical power range: 0.99–1.00.

## Data Availability

The original data and code are available under request.

## Appendix A

**Table A1:** Reliability and convergent validity of the scores obtained in the Blek video game for each minute of play.

	ω	DAT-AR	DAT-SR	DAT-PSA
Third minute Level Reached	0.93	0.39 ***	0.35 ***	0.34 ***
Fourth minute Level Reached	0.95	0.40 ***	0.31 **	0.35 ***
Fifth minute Level Reached	0.96	0.40 ***	0.27 **	0.38 ***
Sixth minute Level Reached	0.97	0.42 ***	0.31 **	0.34 **
Seventh minute Level Reached	0.98	0.42 ***	0.33 **	0.34 **
Eighth minute Level Reached	0.98	0.43 ***	0.36 **	0.33 **
Ninth minute Level Reached	0.99	0.43 ***	0.38 **	0.28 **

Note: To compute ω, it is necessary to have at least three items, which is why we do not include data for the score at two minutes; ** *p* < .01 and *** *p* < .001.

## Appendix B

**Table A2:** Reliability and convergent validity of the scores obtained in the Edge video game for each minute of the game.

	ω	DAT-AR	DAT-SR	DAT-PSA
Third minute Checkpoints Reached	0.94	0.34 **	0.29 **	0.05
Fourth minute Checkpoints Reached	0.96	0.41 **	0.32 **	0.10
Fifth minute Checkpoints Reached	0.96	0.41 **	0.30 **	0.08
Sixth minute Checkpoints Reached	0.97	0.44 **	0.29 **	0.10
Seventh minute Checkpoints Reached	0.98	0.47 **	0.31 **	0.09
Eighth minute Checkpoints Reached	0.99	0.41 **	0.29 **	0.06
Ninth minute Checkpoints Reached	0.99	0.42 **	0.31 **	0.08
Tenth minute Checkpoints Reached	0.99	0.43 **	0.33 **	0.06
Eleventh minute Checkpoints Reached	0.99	0.44 **	0.36 **	0.03

Note: To compute ω, it is necessary to have at least three items, which is why we do not include data for the score at two minutes; ** *p* < .01.

## Appendix C

**Table A3:** Predictive models for the Blek video game per minute of play.

Variable	B	95% CI for B		SE B	β	R^2^	∆R^2^
		(LL–UP)					
First minute							
Step 1						0.24	0.24 ***
Constant	9.72	7.77	11.66	0.98			
Level overcome	1.66	1.06	2.26	0.30	0.49 ***		
Step 2						0.29	0.05 *
Constant	2.48	11.89	29.08	4.33			
Level overcome	1.83	1.13	2.43	0.30	0.54 ***		
Time thinking	−0.24	−0.42	−0.05	0.09	−0.23 *		
Second minute							
Step 1						0.48	0.48 ***
Constant	4.77	2.58	6.96	1.10			
Level overcome	1.89	1.49	2.29	0.20	0.69 ***		
Step 2						0.51	0.03 *
Constant	16.76	6.82	26.71	5.01			
Level overcome	1.85	1.46	2.24	0.20	0.68 ***		
Time thinking	−0.12	−0.22	−0.02	0.05	−0.18 *		
Third minute							
Step 1						0.60	0.60 ***
Constant	3.81	1.92	5.69	0.95			
Level overcome	1.62	1.36	1.89	0.13	0.78 ***		
Step 2						0.66	0.05 ***
Constant	2.94	11.97	29.91	4.52			
Level overcome	1.60	1.35	1.85	0.13	0.77 ***		
Time thinking	−0.12	−0.18	−0.06	0.03	−0.23 ***		
Fourth minute							
Step 1						0.67	0.67 ***
Constant	3.69	2.03	5.34	0.83			
Level overcome	1.37	1.17	1.56	0.10	0.82 ***		
Step 2						0.71	0.03 ***
Constant	16.88	8.8	24.96	4.07			
Level overcome	1.30	1.12	1.49	0.09	0.78 ***		
Time thinking	−0.07	−0.11	−0.03	0.02	−0.19 **		
Fifth minute							
Step 1						0.68	0.68 ***
Constant	4.30	2.74	5.85	0.78			
Level overcome	1.11	0.95	1.26	0.08	0.82 ***		
Step 2						0.72	0.04 ***
Constant	13.03	8.39	17.67	2.33			
Level overcome	1.13	0.99	1.28	0.07	0.84 ***		
Time thinking	−0.04	−0.06	−0.02	0.01	−0.21 ***		
Sixth minute							
Step 1						0.81	0.81 ***
Constant	2.88	1.64	4.12	0.62			
Level overcome	1.12	1.01	1.23	0.05	0.90 ***		
Step 2						0.84	0.02 ***
Constant	1.97	6.53	15.05	2.15			
Level overcome	1.12	1.01	1.22	0.05	0.90 ***		
Time thinking	−0.03	−0.04	−0.01	0.01	−0.16 ***		
Seventh minute							
Step 1						0.88	0.88 ***
Constant	2.30	1.29	3.31	0.51			
Level overcome	1.05	0.97	1.14	0.04	0.94 ***		
Step 2						0.89	0.01 **
Constant	8.52	4.45	12.59	2.05			
Level overcome	1.03	0.96	1.11	0.04	0.92 ***		
Time thinking	−0.02	−0.03	−0.01	0.01	−0.11 **		
Eighth minute							
Step 1						0.93	0.93 ***
Constant	1.50	0.74	2.26	.38			
Level overcome	1.03	0.97	1.08	.03	0.97 ***		
Step 2						0.94	0.00
Constant	5.47	2.05	8.9	1.73			
Level overcome	1.01	0.96	1.07	0.03	0.95 ***		
Time thinking	−0.01	−0.02	0.00	0.00	−0.06 *		
Ninth minute							
Step 1						0.97	0.97 ***
Constant	0.88	0.40	1.36	0.24			
Level overcome	1.00	0.97	1.03	0.02	0.99 ***		

Note: * *p* < .05; ** *p* < .01; *** *p* < .001.

## Appendix D

**Table A4:** Predictive models for the Edge video game per minute of play.

Variable	B	95% CI for B		SE B	β	R^2^	∆R^2^
		(LL–UL)					
First minute							
Step 1						0.37	0.37 ***
Constant	4.05	3.08	5.03	0.49			
Checkpoints overcome	1.31	0.96	1.66	0.18	0.60 ***		
Step 2						0.44	0.07 ***
Constant	40	3.08	4.92	0.46			
Checkpoints overcome	0.84	0.42	1.26	0.21	0.39 ***		
Prisms collected	0.21	0.09	0.33	0.06	0.35 ***		
Second minute						0.52	0.52 ***
Step 1							
Constant	3.83	3.07	4.58	0.38			
Prisms collected	0.40	0.32	0.47	0.04	0.72 ***		
Step 2						0.60	0.08 ***
Constant	3.31	2.58	4.04	0.37			
Prisms collected	0.24	0.14	0.34	0.05	0.44 ***		
Checkpoints overcome	0.51	0.28	0.74	0.11	0.40 ***		
Third minute						0.62	0.62 ***
Step 1							
Constant	4.09	3.50	4.68	0.30			
Checkpoints overcome	0.62	0.52	0.72	0.05	0.78 ***		
Step 2						0.64	0.03 **
Constant	3.73	3.10	4.36	0.32			
Checkpoints overcome	0.39	0.19	0.59	0.10	0.49 ***		
Prisms collected	0.13	0.03	0.23	0.05	0.34 **		
Fourth minute						0.66	0.63 ***
Step 1							
Constant	3.59	2.99	4.20	0.30			
Checkpoints overcome	0.58	0.49	0.66	0.04	0.81 ***		
Step 2						0.68	0.01 *
Constant	3.44	2.83	4.06	0.31			
Checkpoints overcome	0.39	0.18	0.59	0.10	0.54 ***		
Prisms collected	0.09	0.00	0.18	0.05	0.29 *		
Fifth minute						0.72	0.72 ***
Step 1							
Constant	3.38	2.28	3.94	0.28			
Checkpoints overcome	0.51	0.44	0.57	0.03	0.85 ***		
Sixth minute						0.76	0.76 ***
Step 1							
Constant	3.38	2.88	3.89	0.25			
Checkpoints overcome	0.44	0.38	0.49	0.02	0.87 ***		
Step 2						0.77	0.01 *
Constant	5.49	3.61	7.38	0.95			
Checkpoints overcome	0.60	0.45	0.76	0.08	1.21 ***		
Prisms collected	−0.65	−1.20	−0.09	0.29	−0.35 *		
Seventh minute						0.74	0.74 ***
Step 1							
Constant	3.49	2.98	4.01	0.26			
Checkpoints overcome	0.37	0.33	0.42	0.02	0.86 ***		
Eighth minute							
Step 1						0.78	0.78 ***
Constant	3.33	2.85	3.80	0.24			
Checkpoints overcome	0.35	0.31	0.39	0.02	0.89 ***		
Ninth minute							
Step 1						0.80	0.80 ***
Constant	2.75	2.24	3.25	0.25			
Prisms collected	0.18	0.16	0.19	0.01	0.90 ***		
Step 2						0.82	0.02 ***
Constant	1.26	0.28	2.24	0.49			
Prisms collected	0.10	0.05	0.15	0.02	0.50 ***		
Overcome level	0.54	0.23	0.85	0.16	0.42 ***		
Tenth minute							
Step 1						0.86	0.86 ***
Constant	2.59	2.17	3.01	0.21			
Prisms collected	0.17	0.16	0.18	0.01	0.93 ***		
Step 2						0.87	0.00_ns_
Constant	2.74	2.32	3.17	0.21			
Prisms collected	0.09	0.03	0.15	0.03	0.50 **		
Checkpoints overcome	0.15	0.04	0.27	0.06	0.44 *		
Eleventh minute						0.91	0.91 ***
Constant	3.04	2.74	3.37	0.15			
Checkpoints overcome	0.29	0.28	0.31	0.01	0.95 ***		

Note: * *p* < .05; ** *p* < .01; *** *p* < .001.
